# Supplementary material for: Symbiotic bacteria-dependent expansion of MR1-reactive T cells causes autoimmunity in the absence of Bcl11b
Source: Nat Commun. 2022 Nov 14;13:6948. doi: 10.1038/s41467-022-34802-8 (PMC9663695; doi:10.1038/s41467-022-34802-8)
Supplement: Supplementary file 1 — Supplementary Information [file 41467_2022_34802_MOESM1_ESM.pdf]

Supplementary Fig. 1

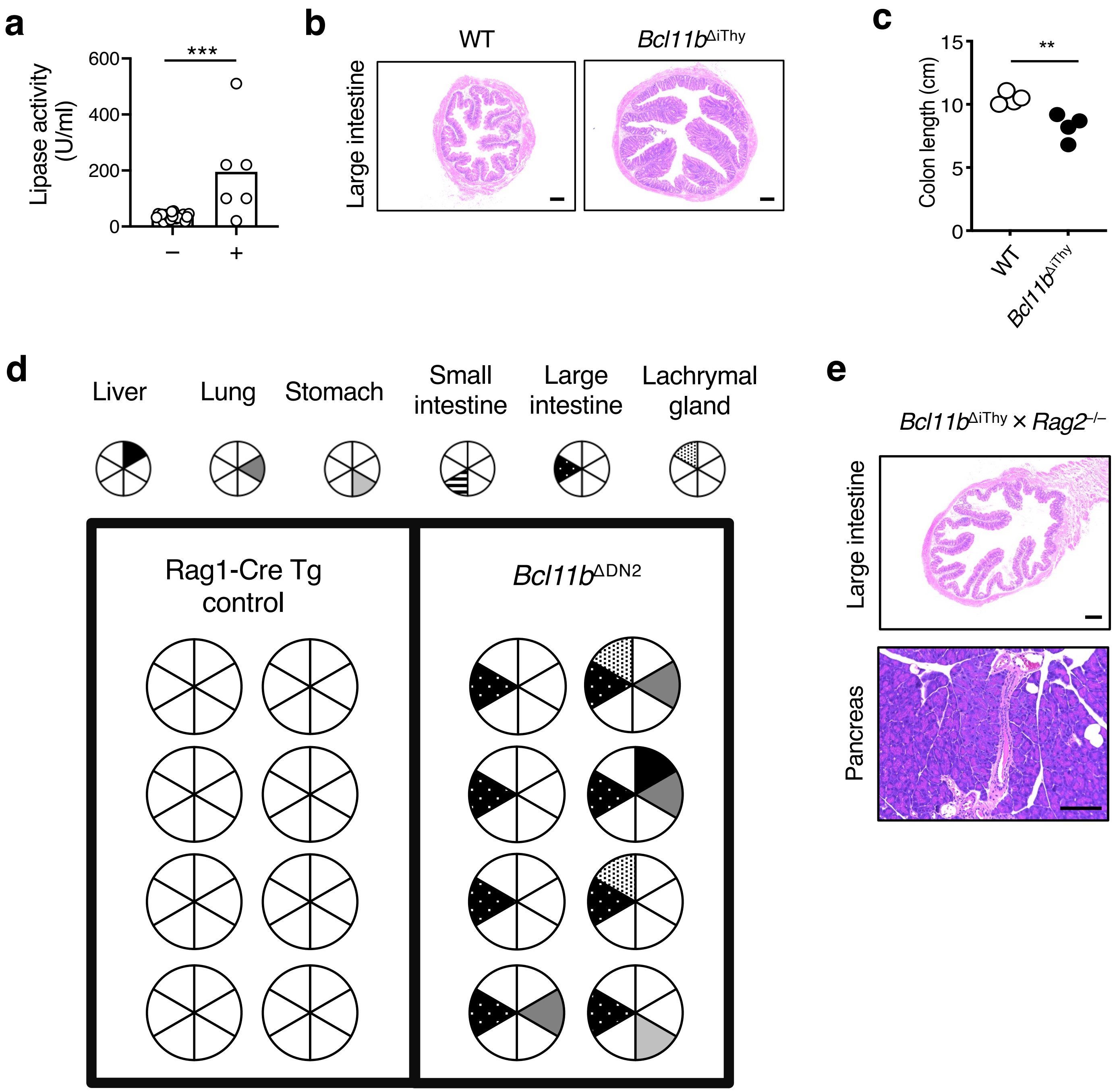

Supplementary Fig. 1. Characterization of chronic inflammation in *Bcl11b*$^{\Delta iThy}$ mice.

a Lipase levels in sera at the 15 weeks of age were compared between *Bcl11b*$^{\Delta iThy}$ mice with (–) or without (+) severe pancreatic inflammation. Statistical significance was determined by unpaired two-tailed Student's t-tests (*** p = 0.0002). **b** Representative H&E staining showing sections of large intestines in WT and *Bcl11b*$^{\Delta iThy}$ mice. Scale bar shows 200 µm. **c** Each plot represents the colon length of a WT or *Bcl11b*$^{\Delta iThy}$ mouse. Statistical significance was determined by unpaired two-tailed Student's t-tests (** p = 0.0079). **d** Summary of inflammation observed in various tissues including liver, lung, stomach, small intestine, large intestine and lachrymal gland. Each circle represents an individual mouse, with the corresponding inflammation in the different tissues indicated above the chart. Scoring was performed following the method used in the previous study (ref. 60). **e** Representative H&E staining showing sections of large intestine and pancreas in *Bcl11b*$^{\Delta iThy}$ × *Rag2*$^{-/-}$ mice. Scale bar shows 200 µm. **c** Data are representative of three independent experiments.

Supplementary Fig. 2

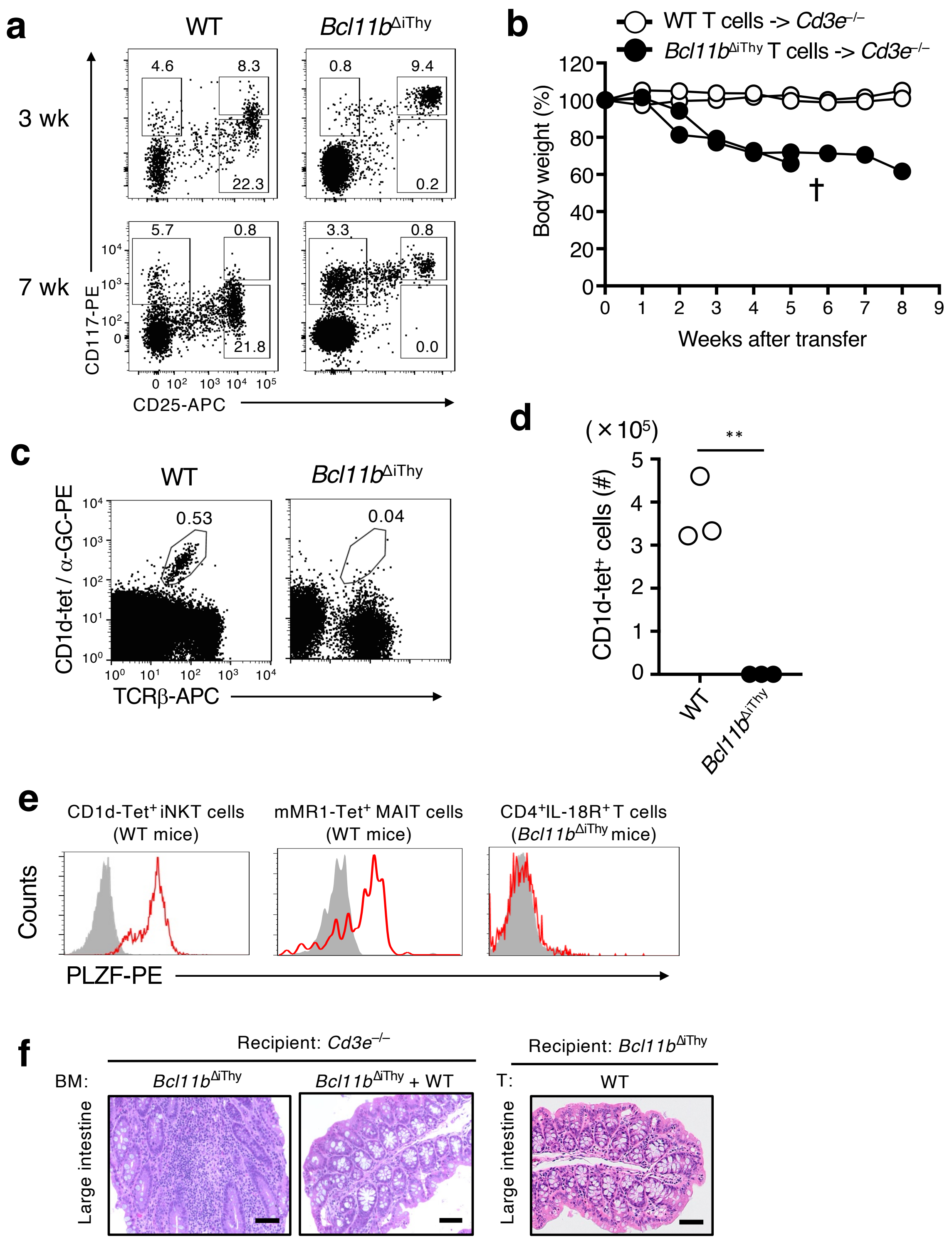

Supplementary Fig. 2. Characterization and pathogenic function of T cells in *Bcl11b*$^{\Delta iThy}$ mice.

a Thymocytes from WT and *Bcl11b*$^{\Delta iThy}$ mice were analyzed after gating on CD4$^-$CD8$^-$ cells. Numbers indicate the percentage of cells within the indicated regions. **b** After *Cd3e*$^{-/-}$ mice were transferred with CD4$^+$ αβ T cells from WT and *Bcl11b*$^{\Delta iThy}$ mice, body weights and survival were monitored. Each line represents body weight changes of the indicated recipient mice. † indicates a recipient mouse that died. **c** Thymocytes of *Bcl11b*$^{\Delta iThy}$ mice were stained with a αGC-loaded CD1d tetramer after gating on total lymphocytes. **d** Each dot represents the absolute number of CD1d-restricted αβ T cells in a mouse of the indicated strain. ** p = 0.0094 by unpaired two-tailed Student's t-tests. **e** Histograms show PLZF expression (red line) in iNKT (aGC-loaded CD1d tetramer$^+$) cells, MAIT (5-OP-RU-loaded mMR1 tetramer$^+$) cells and the indicated cell population from WT or *Bcl11b*$^{\Delta iThy}$ mice as compared with isotype control (gray). **f** Representative H&E staining showing sections of large intestine in recipient mice after transfer of BM cells or T cells. Scale bar shows 60 μm. **b** Data are combined from two independent experiments. **a**, **c-f** Data are representative of three independent experiments.

Supplementary Fig. 3

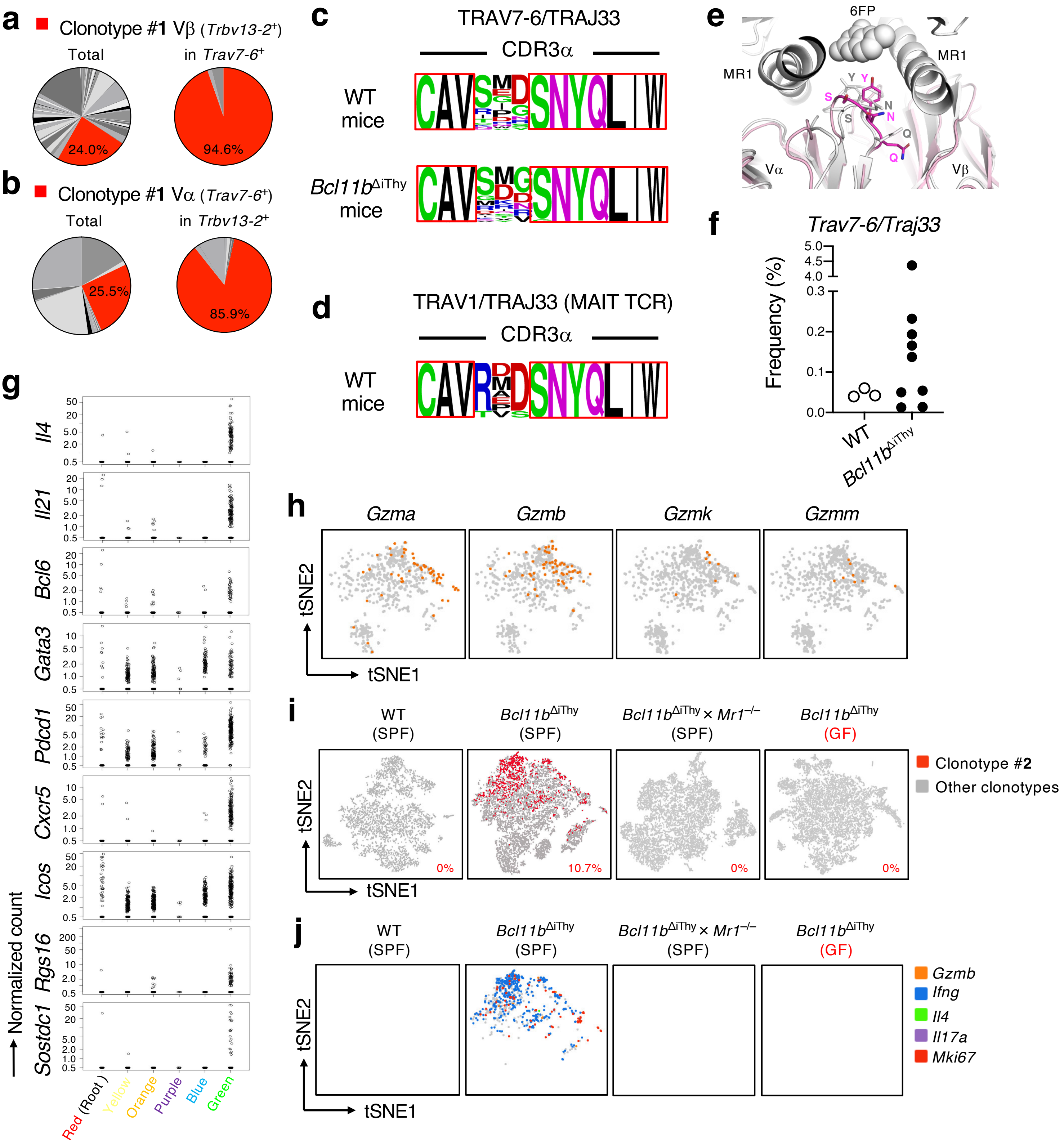

Supplementary Fig. 3. scRNA-seq analysis of αβ T cells in *Bcl11b*$^{\Delta iThy}$ mice.

a, **b** Pie charts show *Trav7-6/Traj33*$^{+}$ cells or *Trbv13-2/Trbj1-1*$^{+}$ cells within annotated αβ T cells or within *Trbv13-2/Trbj1-1*$^{+}$ (**a**) or *Trav7-6/Traj33*$^{+}$ (**b**) cells respectively. CDR3α sequences of annotated αβ T cells were acquired by single cell TCR analysis. **c**, **d** Conserved residues (within the red boxes) in the CDR3α regions of *TRAJ33*$^{+}$ T cells are shown as analyzed with Weblogo (https://weblogo.berkeley.edu/logo.cgi). **e** Putative interaction between TRAJ33 and MR1. The crystal structure of the MAIT TCR-MR1-6-FP ternary complex is superimposed onto the clone #**1** TCRαβ heterodimer (light pink). The positions of four amino acids derived from TRAJ33 (magenta) are overlaid onto the previously reported MAIT TCR (gray) (ref. 17). **f** Each dot represents the frequency of *TRAJ33*$^{+}$ αβ T cells rearranged with *Trav7-6* in a WT or *Bcl11b*$^{\Delta iThy}$ mouse. The frequencies were calculated after bulk RNA-sequencing analysis of sorted αβ T cells from the indicated strains of mice. **g** T$_{FH}$-related gene expression changes in different clusters of clonotype #**1** as shown in Fig. 3 **i**. **h** Colored dots in tSNE plots show clonotype #**1** cells with significantly high expression levels of granzyme family genes in SPF *Bcl11b*$^{\Delta iThy}$ mice. **i** Data from single cell TCR analysis of the indicated mouse strains were projected onto tSNE plots generated by single cell transcriptome analysis. In the tSNE plots, clonotype #**2** (red) and other clonotypes (gray) are shown. Numbers within the plots show the frequency of clonotype #**2**. **j** Colored dots show clonotype #**2** with significantly high expression levels of the indicated genes.

Supplementary Fig. 4

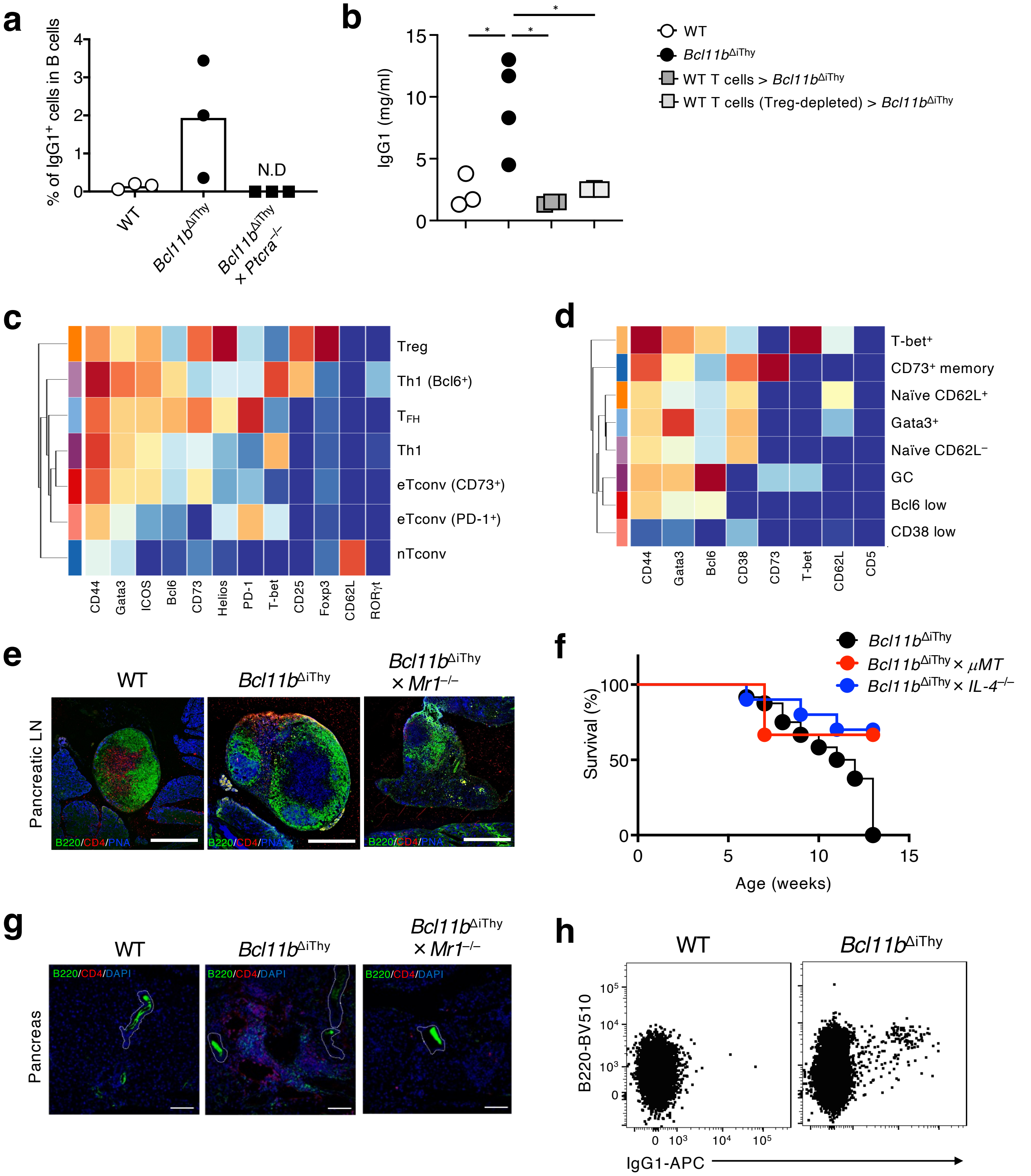

Supplementary Fig. 4. Pathogenic, MR1-dependent IgG production in *Bcl11b*$^{\Delta iThy}$ mice.

a The bar graph shows percentages of IgG1$^+$ B cells within peripheral blood lymphocytes in the indicated strains of mice. N.D. indicates not detected. **b** Productions of total IgG1 in the sera of indicated mice as in Fig. 2 **l**. Asterisks indicate statistical significance determined by unpaired two-tailed Student's t-test (* p = 0.029, 0.0168, 0.0288). **c**, **d** Classification of T (**c**) and B (**d**) cell subsets based on mass cytometric analysis. **c** Heat map graph shows expression profiles of regulatory T cells (Treg), Th1 cells, T_{FH} cells, effector conventional T cells (eTconv), naïve conventional T cells (nTconv). **d** Heat map graph shows expression profiles of indicated B cell subsets. **e** Immunohistochemical analysis of CD4$^+$ αβ T cells (red), B220$^+$ B cells (green) and PNA$^+$ cells (blue) in OCT-embedded pancreatic lymph nodes from the indicated mice. Scale bar: 400 µm. **f** Survival of *Bcl11b*$^{\Delta iThy}$ mice (n = 24), *Bcl11b*$^{\Delta iThy}$ × *µMT* mice (n = 3) and *Bcl11b*$^{\Delta iThy}$ × *Il4*$^{-/-}$ mice (n = 10) monitored for 15 weeks. **g** Immunohistochemical analysis of CD4$^+$ αβ T cells (red) and B220$^+$ B cells (green) in OCT-embedded pancreatic tissues from the indicated strains of mice. Dotted circles indicate pancreatic ducts. Scale bar: 100 µm. **h** IgG1$^+$ B cells in the pancreas of WT and *Bcl11b*$^{\Delta iThy}$ mice. **e**, **g** and **h** Data are representative of two independent experiments. **f** Data are combined from two independent experiment.

Supplementary Fig. 5

a

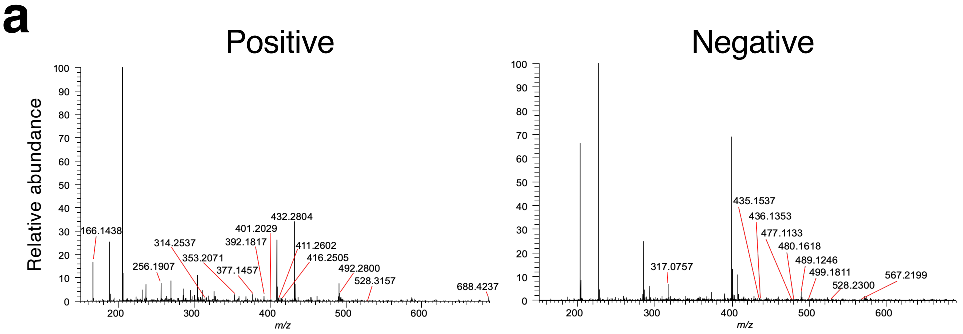

b

ID	Observed precursor ion			Annotation of metabolites by HMDB search[a]
	Adduct ion	*m/z*	Calculated exact mass	HMDB ID (metabolite name)
#54-1	$(M+H)^+$	166.1438	165.1365	–
#54-2	$(M+H)^+$	256.1907	255.1834	–
#54-3	$(M+H)^+$	314.2537	313.2465	–
#54-4	$(M–H)^-$	317.0757	318.0831	–
#54-5	$(M+H)^+$	353.2071	352.2000	–
#54-6	$(M+H)^+$	377.1457	376.1359	HMDB0000244 (riboflavin)
#54-7	$(M+H)^+$	392.1817	391.1745	–
#54-8	$(M+H)^+$	401.2029	400.1958	–
#54-9	$(M+H)^+$	411.2602	410.2531	–
#54-10	$(M+H)^+$	416.2505	415.2430	–
#54-11	$(M+H)^+$	432.2804	431.2733	–
#54-12	$(M–H)^-$	435.1537	436.1587	–
#54-13	$(M–H)^-$	436.1353	437.1417	–
#54-14	$(M–H)^-$	477.1133	478.1204	–
#54-15	$(M–H)^-$	480.1618	481.1679	–
#54-16	$(M–H)^-$	489.1246	490.1310	–
#54-17	$(M+H)^+$	492.2800	491.2746	–
#54-18	$(M–H)^-$	499.1811	500.1884	–
#54-19	$(M+H)^+$	528.3157	527.3097	–
#54-20	$(M–H)^-$	528.2300	529.2372	–
#54-21	$(M–H)^-$	567.2199	568.2262	–
#54-22	$(M+H)^+$	688.4237	687.4171	–

[a] Search criteria: mass error tolerance, exact mass ± 2.5 ppm.

Supplementary Fig. 5. Identification of MR1T cell antigens in the large intestine.
a Full-scan HRMS spectra of fraction #54 in the positive (left) and negative (right) ion mode. **b** Candidate components detected in the fraction #54.

Supplementary Fig. 6

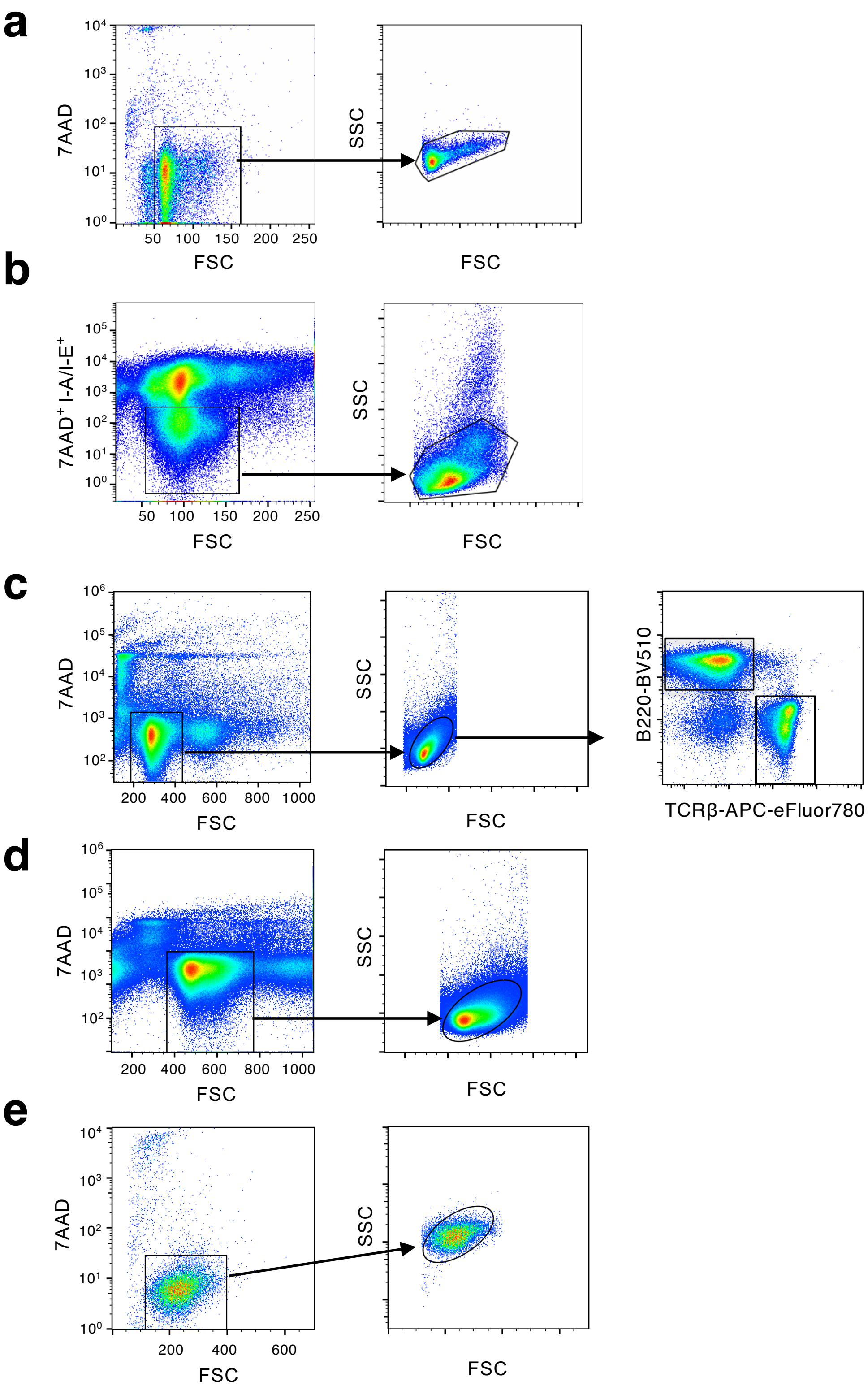

Supplementary Fig. 6. Gating strategy used for flow cytometric analysis.
Gating strategies for flow cytometric analysis of T cells in the thymus (**a**), the spleen (**b-d**) and T cell hybridomas (**e**) are shown. After excluding 7-Aminoactinomycin D (7AAD)$^{+}$ dead cells (**a**, **c**, **d**, **e**) or 7AAD^{+} I-A/I-E^{+} cells (**b**) (left), lymphocytes or hybridomas are identified based on parameters of FSC and SSC (second plots from the left). **c** Viable lymphocytes are further classified into TCRβ^{+} T cells and B220^{+} B cells (right).

Supplementary Table 1. Antibodies for mass cytometric analysis.

**Antibodies for cellular barcoding**					
**Metal**	**Antigen**	**Clone**	**Cat. No.**	**Manufacturer**	**Titration**
113In	CD45	30-F11	103141	Biolegend	100
115In	CD45	30-F11	103141	Biolegend	100
194Pt	CD45	30-F11	103141	Biolegend	100
198Pt	CD45	30-F11	103141	Biolegend	100

**Antibodies for surface staining**					
**Metal**	**Antigen**	**Clone**	**Cat No.**	**Manufacturer**	**Titration**
141Pr	Ly6G	1A8	127637	Biolegend	100
142Nd	CD5	53-7.3	100619	Biolegend	100
143Nd	CD103	2E7	121402	Biolegend	100
145Nd	CD69	H1.2F3	104533	Biolegend	100
147Sm	KLRG1	2F1	16-5893-85	Thermo Fisher Scientific	100
148Nd	CD11b	M1/70	101249	Biolegend	100
149Sm	Ly6C	HK1.4	128039	Biolegend	200
150Nd	CD73	TY/11.8	127202	Biolegend	200
151Eu	CD25	PC61	14-0251-85	Thermo Fisher Scientific	200
152Sm	CD3e	145-2C11	100345	Biolegend	100
153Eu	CD39	24DMS1	14-0391-82	Thermo Fisher Scientific	100
154Sm	OX40	OX-86	14-1341-82	Thermo Fisher Scientific	100
155Gd	PD-1	RMP1-30	109113	Biolegend	100
160Gd	CD62L	MEL-14	104443	Biolegend	400
162Dy	TIM3	RMT3-23	3162029B	Standard BioTools	100
163D	CD8a	53-6.7	100755	Biolegend	100
166Er	ICOS	C398.4A	14-9949-82	Thermo Fisher Scientific	100
168Er	CD8a	53-6.7	100755	Biolegend	100
169Tm	TCRβ	H57-597	109235	Biolegend	100
170Er	NK1.1	PK136	108743	Biolegend	100
171Yb	CD44	IM7	103051	Biolegend	400
172Yb	CD4	RM4-5	100561	Biolegend	200
173Yb	GITR	DTA-1	126321	Biolegend	100
175Lu	CD38	90	3175014B	Standard BioTools	200
176Yb	B220	RA3-6B2	103249	Biolegend	200
209Bi	MHCII	M5/114.15.2	3209006B	Standard BioTools	100

**Antibodies for intracellular staining**					
**Metal**	**Antigen**	**Clone**	**Cat No.**	**Manufacturer**	**Titration**
146Nd	Helios	22F6	137202	Biolegend	100
158Gd	Foxp3	FJK-16s	14-5773-82	Thermo Fisher Scientific	100
159Tb	RORgt	B2D	3159019B	Standard BioTools	100
161Dy	T-bet	4B10	644825	Biolegend	100
164Dy	CTLA-4	UC10-4B9	14-1522-82	Thermo Fisher Scientific	100
165Ho	BCL6	K112-91	561520	BD Biosciences	200
167Er	Gata-3	L50-823	558686	BD Biosciences	200

Supplementary Table 2. Data collection and refinement statistics of clone #**1** ectodomain

Data collection statistics	Beamline	Photon Factory BL-1A
	Space group	$P2_12_12_1$
	Cell constant (Å)	a=64.9, b=73.8, c=170.3
	Resolution (Å)*	48.72-3.00 (3.16-3.00)
	Rmerge (%)*	18.5 (97.3)
	Rpim (%)*	7.5 (43.0)
	Completeness (%)*	99.9 (99.9)
	$<I/\sigma I>$*	9.7 (1.8)
	Redundancy*	6.9 (6.1)
Refinement statistics	R (%)	22.5
	Rfree (%)	31.1
Root mean square deviations from ideal values	Bond length (Å)	0.011
	Bond angle (°)	1.34
Ramachandran plot	Favored/Allowed (%)	86.6/13.2
	Outlier (%)	0.2
	Rotamer outlier (%)	0

*Values in the parenthesis were the highest resolution shells.
