# Supplementary material for: Symbiotic bacteria-dependent expansion of MR1-reactive T cells causes autoimmunity in the absence of Bcl11b
Source: Nat Commun. 2022 Nov 14;13:6948. doi: 10.1038/s41467-022-34802-8 (PMC9663695; doi:10.1038/s41467-022-34802-8)
Supplement: Supplementary file 3 — Description of Additional Supplementary Files [file 41467_2022_34802_MOESM3_ESM.pdf]

Description of Additional Supplementary Files

Title: **Supplementary Code**
Description: Supplementary_code.txt: Modified plotGeneCount module in the tradeSeq package enabling to change color and position of scale bar.
Supplementary_data.RData: Gene expression data which is an input to the function of Supplemental_code.txt
