# Supplementary material for: Symbiotic bacteria-dependent expansion of MR1-reactive T cells causes autoimmunity in the absence of Bcl11b
Source: Nat Commun. 2022 Nov 14;13:6948. doi: 10.1038/s41467-022-34802-8 (PMC9663695; doi:10.1038/s41467-022-34802-8)
Supplement: Supplementary file 5 — Reporting Summary [file 41467_2022_34802_MOESM5_ESM.pdf]

nature portfolio

Corresponding author(s): Sho Yamasaki

Last updated by author(s): Sep 30, 2022

Reporting Summary

Nature Portfolio wishes to improve the reproducibility of the work that we publish. This form provides structure for consistency and transparency in reporting. For further information on Nature Portfolio policies, see our Editorial Policies and the Editorial Policy Checklist.

Statistics

For all statistical analyses, confirm that the following items are present in the figure legend, table legend, main text, or Methods section.

n/a	Confirmed	
☐	☒	The exact sample size (*n*) for each experimental group/condition, given as a discrete number and unit of measurement
☐	☒	A statement on whether measurements were taken from distinct samples or whether the same sample was measured repeatedly
☐	☒	The statistical test(s) used AND whether they are one- or two-sided<br>*Only common tests should be described solely by name; describe more complex techniques in the Methods section.*
☒	☐	A description of all covariates tested
☒	☐	A description of any assumptions or corrections, such as tests of normality and adjustment for multiple comparisons
☐	☒	A full description of the statistical parameters including central tendency (e.g. means) or other basic estimates (e.g. regression coefficient) AND variation (e.g. standard deviation) or associated estimates of uncertainty (e.g. confidence intervals)
☐	☒	For null hypothesis testing, the test statistic (e.g. *F*, *t*, *r*) with confidence intervals, effect sizes, degrees of freedom and *P* value noted<br>*Give P values as exact values whenever suitable.*
☒	☐	For Bayesian analysis, information on the choice of priors and Markov chain Monte Carlo settings
☒	☐	For hierarchical and complex designs, identification of the appropriate level for tests and full reporting of outcomes
☒	☐	Estimates of effect sizes (e.g. Cohen's *d*, Pearson's *r*), indicating how they were calculated

Our web collection on statistics for biologists contains articles on many of the points above.

Software and code

Policy information about availability of computer code

Data collection	FACS data were collected using FACS Verse (BD Biosciences) and Beckman Coulter Kaluza (Beckman Coulter). Mass cytometry data were collected using a Helios mass cytometer (Standard BioTools).
Data analysis	Flow cytometric analysis was performed with FlowJo (version 10.6.1). Prism (version 9.0.2) was used for statistical analysis.<br>Mass spectrometry data analysis was performed by CytoBank software. Clustering was performed by FlowSOM (version 3) within the CATALYST Bioconductor package.<br>Single cell analysis was performed by softwares as follows : Loupe VDJ browser (version 3.0.0) and Loupe Browser (version 4.2.0) for VDJ and transcriptome analysis ; slingshot (version 1.4.0) and tradeSeq (version 1.0.0) within Bioconductor packages for pseudotime analysis ; FlowSOM (version 3) and edgeR (version 3.38.4) for masscytometry analysis.<br>The customized plotGeneCount script and source data for Fig. 3k were provided as "Supplemental_code.txt" and "Supplemental.RData", respectively. The script code provided in the supplementary material is available under the MIT License (Copyright 2022, Takahide Hayano). The original code was included in the package "tradeSeq" (https://bioconductor.org/packages/release/bioc/html/tradeSeq.html) which is distributed under the MIT License (Copyright 2019, Koen Van den Berge; Hector Roux de Bezieux). See https://opensource.org/licenses/mit-license.php for the full text of the MIT license.

For manuscripts utilizing custom algorithms or software that are central to the research but not yet described in published literature, software must be made available to editors and reviewers. We strongly encourage code deposition in a community repository (e.g. GitHub). See the Nature Portfolio guidelines for submitting code & software for further information.

Data

Policy information about availability of data

All manuscripts must include a data availability statement. This statement should provide the following information, where applicable:

- Accession codes, unique identifiers, or web links for publicly available datasets
- A description of any restrictions on data availability
- For clinical datasets or third party data, please ensure that the statement adheres to our policy

Bulk RNA-sequencing and single-cell RNA-sequencing data have been deposited at the DNA Data Bank of Japan (DDBJ) database under the accession number DRA011320 [https://ddbj.nig.ac.jp/resource/bioproject/PRJDB10983]. Structure factor and 3D coordinates of clone #1 TCR ectodomain have been deposited in the Protein Data Bank under the accession code, 7F5K [https://www.rcsb.org/structure/7F5K].

Field-specific reporting

Please select the one below that is the best fit for your research. If you are not sure, read the appropriate sections before making your selection.

☒ Life sciences ☐ Behavioural & social sciences ☐ Ecological, evolutionary & environmental sciences

For a reference copy of the document with all sections, see nature.com/documents/nr-reporting-summary-flat.pdf

Life sciences study design

All studies must disclose on these points even when the disclosure is negative.

Sample size	Information such as age, gender of mice used in this study was shown in the Figure legends section. For single experiment, we always prepare more than three biological animals in each group.
Data exclusions	No data were excluded.
Replication	For experiments with high variability, we used n>5. Other experiments were done at least with n>3. We confirmed that all attempts at replicon are successful.
Randomization	No formal randomization techniques were applied. Groups were generated based on their genotypes or disease symptoms. Samples were allocated randomly to experiments and processed in an arbitrary order.
Blinding	For all analysis using mice, the investigators were blinded to group allocation during data collection.

Reporting for specific materials, systems and methods

We require information from authors about some types of materials, experimental systems and methods used in many studies. Here, indicate whether each material, system or method listed is relevant to your study. If you are not sure if a list item applies to your research, read the appropriate section before selecting a response.

Materials & experimental systems

n/a	Involved in the study	
☐	☒	Antibodies
☐	☒	Eukaryotic cell lines
☒	☐	Palaeontology and archaeology
☐	☒	Animals and other organisms
☒	☐	Human research participants
☒	☐	Clinical data
☒	☐	Dual use research of concern

Methods

n/a	Involved in the study	
☒	☐	ChIP-seq
☐	☒	Flow cytometry
☒	☐	MRI-based neuroimaging

Antibodies

Antibodies used	For Flow cytometric analysis, αGC-loaded CD1d tetramers were used following manufacturer's instruction (Cat No. TS-MCD-1, Medical & Biological Laboratories co., LTD). For staining of mouse MAIT cells, APC-labeled mouse MR1 tetramer provided by NIH Tetramer core facility was used. FITC-conjugated anti-mouse (m) CD45.1 (A20, 1:40 dilution, Cat No.110705), anti-mCD45.2 (104, 1:100 dilution, Cat No.109805) mAbs, PE-conjugated anti-mCD5 (53-7.3, 1:100 dilution, Cat No.100607), anti-mCD8α (53-6.7, 1:80 dilution, Cat No.100707), anti-mCD40L/CD154 (MR1, 1:100 dilution, Cat No.106505), anti-mCD117 (2B8, 1:40 dilution, Cat No.105807), and anti-MR1 (26.5, 1:40 dilution, Cat No.361105) mAbs, PECy7-conjugated anti-mCD4 (RM4-5, 1:200 dilution, Cat No.100527) mAb, PerCP-Cy5.5-conjugated anti-mI-A/I-E (M5/114.15.2, 1:200 dilution, Cat No.107623) and anti-mCD44 (IM7, 1:200 dilution, Cat No.103031) mAbs, APC-conjugated anti-mTCRβ (H57-597, 1:40 dilution, Cat No.109211), anti-mCD4 (RM4-5, 1:100

dilution, Cat No.100515), anti-mIL-18Rα (A17071D, 1:100 dilution, Cat No.157905), anti-mCD25 (PC61, 1:200 dilution, Cat No.102011), anti-mCD44 (IM7, 1:200 dilution, Cat No.103011), anti-mCD45.2 (104, 1:40 dilution, Cat No.109813) and anti-mCD62L (MEL-14, 1:100 dilution, Cat No.104411) mAbs, AF700-conjugated mCD8 (53-6.7, 1:100 dilution, Cat No.100729) mAb, APCCy7-conjugated mTCRβ (H57-597, 1:40 dilution, Cat No.109219) mAb, BV421-conjugated mTCRβ (H57-597, 1:40 dilution, Cat No.109229) and mCD8α (53-6.7, 1:80 dilution, Cat No.100737) mAbs, BV510-conjugated mB220 (RA3-6B2, 1:100 dilution, Cat No.103247) and anti-mCD4 (RM4-5, 1:100 dilution, Cat No.100553) mAbs were purchased from BioLegend. FITC-conjugated anti-mIgG1 (A85-1, 1:50 dilution, Cat No.553443) mAb was purchased from BD Biosciences. PE-conjugated anti-mIL-18R (P3TUNYA, 1:80, Cat No.12-5183-82) and APC-eFluor780 conjugated anti-mTCRβ (H57-597, 1:20, Cat No.47-5961-82) mAbs were purchased from Thermo Fisher Scientific.
For histological analysis, antibodies against mIgG1 (0.5 μg/ml, A85-1, Cat. No.553443, BD Bioscience), mCD4 (10 μg/ml, GK1.5, Cat. No.100416, BioLegend), B220 (5 μg/ml, RA3-6B2, Cat. No.103225, BioLegend), biotinylated PNA (20 μg/ml, Cat. No.BK-1000, Vector laboratories), Alexa Fluor 647-conjugated Streptavidin (1 μg/ml, Cat. No.405237, BioLegend) were used.
For Mass cytometry analysis, anti-mCD45 (30-F11, 1:100 dillution, Cat. No.103141), anti-mLy6G (1A8, 1:100 dillution, Cat. No.127637), anti-mCD5 (53-7.3, 1:100 dillution, Cat. No.100619), anti-mCD103 (2E7, 1:100 dillution, Cat. No.121402), anti-mCD69 (H1.2F3, 1:100 dillution, Cat.No.104533), anti-mCD11b (M1/70, 1:100 dillution, Cat. No.101249), anti-mLy6C (HK1.4, 1:200 dillution, Cat. No.128039), anti-mCD73 (TY/11.8, 1:200 dillution, Cat. No.127202), anti-mCD3e (145-2C11, 1:100 dillution, Cat. No.100345), anti-mPD1 (RMP1-30, 1:100 dillution, Cat. No.109113), anti-mCD62L (MEL-14, 1:400 dillution, Cat. No.104443), anti-mCD8a (53-6.7, 1:100 dillution, Cat. No.100755), anti-mTCRβ (H57-597, 1:100 dillution, Cat. No.109235), anti-mNK1.1 (PK136, 1:100 dillution, Cat. No.108743), anti-mCD44 (IM7, 1:400 dillution, Cat. No.103051), anti-mCD4 (RM4-5, 1:200 dillution, Cat. No.100561), anti-mGITR (DTA-1, 1:100 dillution, Cat. No.126321), anti-B220 (RA3-6B2, 1:200 dillution, Cat. No.103249) and anti-mHelios (22F6, 1:100 dillution, Cat. No.137202) mAbs were purchased from Biolegend. Anti-mKLRG1 (2F1, 1:100 dillution, Cat. No.16-5893-85), anti-mCD25 (PC61, 1:200 dillution, Cat. No.14-0251-85), anti-mCD39 (24DMS1, 1:100 dillution, Cat. No.14-0391-82), anti-mOX40 (OX-86, 1:100 dillution, Cat. No.14-1341-82), anti-mICOS (C398.4A, 1:100 dillution, Cat. No.14-9949-82), anti-mFoxp3 (FJK-16s, 1:100 dillution, Cat.No.14-5773-82) and anti-mCTLA4 (UC10-4B9, 1:100 dillution, Cat. No.14-1522-82) mAbs were purchased from Thermo Fisher Scientific. Anti-mTIM3 (RMT3-23, 1:100 dillution, Cat. No.3162029B), anti-mCD38 (90, 1:200 dillution, Cat. No.3175014B), anti-mMHCII (M5/114.15.2, 1:100 dillution, Cat. No.3209006B) and anti-mRORgt (B2D, 1:100 dillution, Cat. No.3159019B) mAbs were purchased from Standard BioTools. Anti-mBCL6 (K112-91, 1:200 dillution, Cat. No.561520) and anti-mGata-3 (L50-823, 1:200 dillution, Cat. No.558686) mAbs were purchased from BD Biosciences.

Validation

Validation information is found in each manufacture's websites.
Medical & Biological Laboratories co., LTD : https://ruo.mbl.co.jp/bio/dtl/T/index.html?pcd=TS-MCD-1
NIH Tetramer core facility : https://tetramer.yerkes.emory.edu/reagents/mr1
Biolegend : https://www.biolegend.com/ja-jp/flow-cytometry
BD Biosciences : https://www.bdbiosciences.com/en-us/products/reagents
Thermo Fisher Scientific : https://www.thermofisher.com/jp/ja/home/life-science/antibodies/ebioscience.html
Standard BioTools : https://www.standardbiotools.com/products-services/kits-reagents-and-accessories/cytometry

Eukaryotic cell lines

Policy information about cell lines

Cell line source(s)	NIH3T3 cells (Cat. No.CRL1658, ATCC)
Authentication	MR1-overexpressing NIH3T3 cells are not autheticated.
Mycoplasma contamination	We confirmed that cell lines are negative for Mycoplasma contamination.
Commonly misidentified lines (See ICLAC register)	We do not use misidentified lines in this study.

Animals and other organisms

Policy information about studies involving animals; ARRIVE guidelines recommended for reporting animal research

Laboratory animals	C57BL/6 mice, MR1-deficeint mice, Rag1Cre/+ mice, Bcl11b flox/flox mice, Il4 GFP/GFP mice, mMT mice were used in this study.
Wild animals	We did not use wild animals in this study.
Field-collected samples	We did not use field-collected samples in this study.
Ethics oversight	All animal protocols were approved by the Committee of Ethics on Animal Experiments in the Faculty of Medicine of Kyushu University, Science Research Center of Institute of Life Science and Medicine of Yamaguchi University and Research Institute for Microbial Diseases of Osaka University.

Note that full information on the approval of the study protocol must also be provided in the manuscript.

Flow Cytometry

Plots

Confirm that:

☒ The axis labels state the marker and fluorochrome used (e.g. CD4-FITC).

☒ The axis scales are clearly visible. Include numbers along axes only for bottom left plot of group (a 'group' is an analysis of identical markers).

☒ All plots are contour plots with outliers or pseudocolor plots.

☒ A numerical value for number of cells or percentage (with statistics) is provided.

Methodology

Sample preparation	Cells from spleen, thymus and mesenteric lymph node were obtained by mechanical disruption. After lysing red blood cells, cells were passed through a cell strainer to obtain single cell suspension. Isolated cells were stained with antibodies and/or tetramers, as described in the Methods section.
Instrument	BD FACS Aria III and Sony SH800s (for cell sorting or analysis), BD FACSCalibur, BD FACSVerse and Beckman Coulter Gallios Flow Cytometer (for analysis).
Software	FlowJo (version 10.6.1)
Cell population abundance	Post-sort fractions were >98% pure in experiments.
Gating strategy	After preparation of single cell suspensions from various tissues, dead cells or debris were excluded by FSC-A/SSC-A and 7AAD+ gates. In the gate on viable cells, T cells and B cells were identified by pan-markers. Additional markers for each population used in this study was described in the figures and figure legends.

☐ Tick this box to confirm that a figure exemplifying the gating strategy is provided in the Supplementary Information.
